# Age-related differences in the temporal dynamics of spectral power during memory encoding

**DOI:** 10.1371/journal.pone.0227274

**Published:** 2020-01-16

**Authors:** M. Karl Healey, Michael J. Kahana

**Affiliations:** 1 Department of Psychology, Michigan State University, East Lansing, MI, United States of America; 2 Department of Psychology, University of Pennsylvania, Philadelphia, PA, United States of America; Nathan S Kline Institute, UNITED STATES

## Abstract

We examined oscillatory power in electroencephalographic recordings obtained while younger (18-30 years) and older (60+ years) adults studied lists of words for later recall. Power changed in a highly consistent way from word-to-word across the study period. Above 14 Hz, there were virtually no age differences in these neural gradients. But gradients below 14 Hz reliably discriminated between age groups. Older adults with the best memory performance showed the largest departures from the younger adult pattern of neural activity. These results suggest that age differences in the dynamics of neural activity across an encoding period reflect changes in cognitive processing that may compensate for age-related decline.

## Introduction

Memory impairments are among the most common complaints of older adults [1]. Much effort has been devoted to identifying the neurocognitive causes of age-related memory decline [2, 3]. But one potential source of age differences has received little attention: the ability to sustain encoding processes across a series of events or items that unfold over time [4]. For example, the people you meet during a job interview, the grocery list your spouse dictates over the phone, or which of your medications you have already taken today.

Researchers have studied this aspect of memory using the free recall task, in which subjects study a list of sequentially presented items (e.g., words) and then recall the items in any order. The nature of the encoding processes in which subjects engage changes from item-to-item as the list is studied [5]. These changes unfold in the brain without any obvious behavioral correlates—they can only be inferred from which items are subsequently remembered and forgotten. Perhaps for this reason, most cognitive aging theories are silent about the contribution of encoding dynamics to memory impairments [3, 6–8].

We argue, however, that there are two general categories of item-to-item changes in cognitive processing that are likely to show age differences. The first category includes processes that become less efficient as the list progresses with time due to fatigue [9]. The second category includes processes that ramp up as the list goes on, such as rehearsing early items in the list [10]. Although differences in such processes are difficult to detect from behavior, they should leave a signature in how neural activity changes while studying a list. Indeed, recent evidence suggests that long periods of cognitive engagement are associated with specific neural substrates [11].

We sought to provide an initial test of the hypothesis that there are age differences in the dynamics of neural activity across the encoding period of a free recall list and that these processing differences may either contribute to, or compensate for, age-related memory impairment. Our approach was to examine electroencephalographic (EEG) recordings taken while subjects study lists for free recall. We analyzed the data by converting raw EEG into the frequency domain and examining how spectral power changes across time during the study period. We then tested for age differences in these across-time changes in spectral power. Finally, we tested whether the neural age differences could predict behavioral age differences in memory performance.

## Materials and methods

This study was approved by the University of Pennsylvania Institutional Review Board. Written informed consent was obtained from all subjects. The data are from the Penn Electrophysiology of Encoding and Retrieval Study (PEERS), an ongoing project aiming to assemble a large database on memory ability in older and younger adults.

### Subjects

Subjects were recruited for PEERS through a two–stage process. First, we recruited right-handed native English speakers for a single session. Older adults were pre-screened for signs of pathology using a detailed medical history and the Short Blessed Test [12]. The second stage of recruitment focused only on subjects who did not make an excess of eye movements during item presentation epochs of the introductory session and had a recall probability of less than 0.8. These criteria were used to reduce the chance that subjects’ recall performance would reach ceiling across the seven sessions of Experiment 1. Approximately half of the subjects recruited for the preliminary session satisfied these criteria and agreed to participate in the full study. The present analyses are based on the 172 younger adults (age 17–30) and 36 older adults (age 61-85 years) who had entered the full study and had completed Experiment 1 of PEERS as of September 2015. See [9] for details on these samples.

### PEERS experiment

The analyses reported here focus on the free recall data from PEERS Experiment 1, which consisted of seven sessions each of which included 16 free recall lists. For each list, 16 words were presented one at a time on a computer screen followed by an immediate free recall test. Each session ended with a recognition test. The first session and half of the remaining sessions were randomly chosen to include a final free recall test before recognition, in which participants recalled words from any of the lists from the session. The recognition data are not examined here, but details on these data can be found in prior publications [9].

Each word was accompanied by a cue to perform one of two judgment tasks (“Will this item fit into a shoebox?” or “Does this word refer to something living or not living?”) or no encoding task. The current task was indicated by the color and typeface of the presented item. There were three conditions: no-task lists (subjects did not have to perform judgments with the presented items), single-task lists (all items were presented with the same task), and task-shift lists (items were presented with either task). The first two lists were task-shift lists, and each list started with a different task. The next 14 lists contained 4 no-task lists, 6 single-task lists (3 of each of the task), and 4 task-shift lists. List and task order were counterbalanced across sessions and subjects.

Each stimulus was drawn from a pool of 1638 words. Lists were constructed such that varying degrees of semantic relatedness occurred at both adjacent and distant serial positions. Semantic relatedness was determined using the Word Association Space (WAS) model [13]. WAS similarity values were used to group words into four similarity bins (high similarity: *cosθ* between words > 0.7; medium-high similarity, 0.4 < *cosθ* < 0.7; medium-low similarity, 0.14 < *cosθ* < 0.4; low similarity, *cosθ* < 0.14). Two pairs of items from each of the four groups were arranged such that one pair occurred at adjacent serial positions and the other pair was separated by at least two other items. This semantic manipulation has been analyzed elsewhere [14] and will not be considered here as it is not relevant to our present focus and the distribution of these pairs across serial positions ensures that they are not confounded with age differences in neural dynamics. For each list, there was a 1500 ms delay before the first word appeared on the screen. Each item was on the screen for 3000 ms, followed by jittered (i.e., variable) inter-stimulus interval of 800-1200 ms (uniform distribution). If the word was associated with a task, subjects indicated their response via a keypress. After the last item in the list, there was a jittered delay of 1200-1400 ms, after which a tone sounded, a row of asterisks appeared, and the subject was given 75 seconds to attempt to recall aloud any of the just-presented items.

### Electrophysiological recordings and data processing

We used Netstation to record EEG from Geodesic Sensor Nets (Electrical Geodesics, Inc.) with 129 electrodes digitized at 500 Hz by either the Net Amps 200 or 300 amplifier and referenced to Cz. Recordings were then rereferenced to the average of all electrodes except those with high impedance or poor scalp contact. We identified electrodes that likely had high impedance or poor scalp contact by dividing the epochs of interest into 1000 ms bins and excluding those electrodes for which the range was above 200 *μV* in more than 20% of bins. To eliminate electrical line noise, a fourth order 2 Hz stopband butterworth notch filter was applied at 60 Hz.

To correct artifacts such as eye blinks or electrodes with poor contacts, we used independent component analysis (ICA [15]) and an artifact detection/correction algorithm based on [16]. Manual identification of artifactual independent components (IC) can be unreliable [16] and would be impractical given the number and length of sessions in the current study. Therefore, we used an automatic artifact correction algorithm [16]. The algorithm starts with raw EEG. For each channel, several statistics were used to identify channels with severe artifacts. First, electrodes should be moderately correlated with other electrodes due to volume conduction, thus the mean correlation between the channel and all other channels was calculated, and these means were z-scored across electrodes. Channels with z-scores less than -3 were rejected. Second, electrodes with very high or low variance across a session are likely dominated by noise or have poor contact with the scalp; therefore, the variance was calculated for each electrode and z-scored across electrodes. Electrodes with a |*z*| ≥ 3 were rejected. Finally, we expect many electrical signals to be autocorrelated, but signals generated by the brain versus noise likely have different forms of autocorrelation. Therefore, the Hurst exponent, which is a measure of long-range autocorrelation was calculated for each electrode and electrodes with a |*z*| ≥ 3 were rejected. Electrodes that were marked as bad by this procedure were interpolated using EEGLAB’s [17] spherical spline interpolation algorithm. The median number of electrodes interpolated per session was 1 and the maximum number interpolated for any session was 10. The maximum number of ICs that can be reliably estimated depends on the number of samples recorded for each channel. We extracted $c=floor(\sqrt{L/k})$ ICs where *L* is the number of samples in the session and *k* is a constant set to 25 (for a discussion of *k*, see [16, 18]) or the number of non-interpolated channels, whichever was smaller. We then ran EEGLAB’s implementation of infomax ICA [15, 17] on the first *c* principal components of the EEG matrix to decompose it into ICs.

ICs that capture blinks or saccades should be highly correlated with the raw signal from the EOG electrodes. Therefore, for each IC we computed the absolute value of its correlation with each of the six EOG electrodes, retained the maximum of those values and z-scored the maximum correlations across ICs. ICs with |*z*| ≥ 3 were rejected. ICs that capture artifacts isolated to single electrodes (e.g., an electrode shifting or “popping off”) should have high weights for the implicated electrodes but low weights for other electrodes. To identify such ICs, we calculated the kurtosis of the weights across electrodes and excluded any IC with a z-score above +3. Finally, ICs capturing white noise should have a nearly flat power spectrum (versus the 1/*f* spectrum expected for neural signals). Therefore, we calculated the absolute value of the slope of the power spectrum for the frequencies included in the analyses (2–200 Hz) and rejected ICs with *z* ≥ −3 (i.e., the ones closest to zero slope). Rejected ICs were removed from the matrix and the remaining IC activation time courses were projected back into electrode space. All subsequent analyses were carried out on this corrected EEG data.

To compute spectral power, the corrected EEG data time series for an entire session was convolved with Morlet wavelets (wave number = 6) at each of 60 frequencies logarithmically spaced between 2 Hz and 200 Hz. The resulting power time series were downsampled to 10 Hz. We then defined encoding events by extracting the time period from -200 ms to 3000 ms relative to each item’s presentation. For each frequency, a subject’s raw power values were z-scored across encoding events separately for each session and each encoding task (no-task, single-task, and task-shift) to remove the effects of these variables which are known to affect power [19]. Z-scored power was then averaged across the -200 ms to 3000 ms encoding interval to provide one power value for each study event.

## Results

To test for age differences in the dynamics of encoding, we examined EEG signals recorded while the subjects studied the lists. We analyzed spectral power derived from the EEG signals as past research has shown that effective memory encoding is correlated with spectral power in specific frequency bands [20] and that spectral power shows reliable age differences during memory tasks [2].

**Fig 1:**
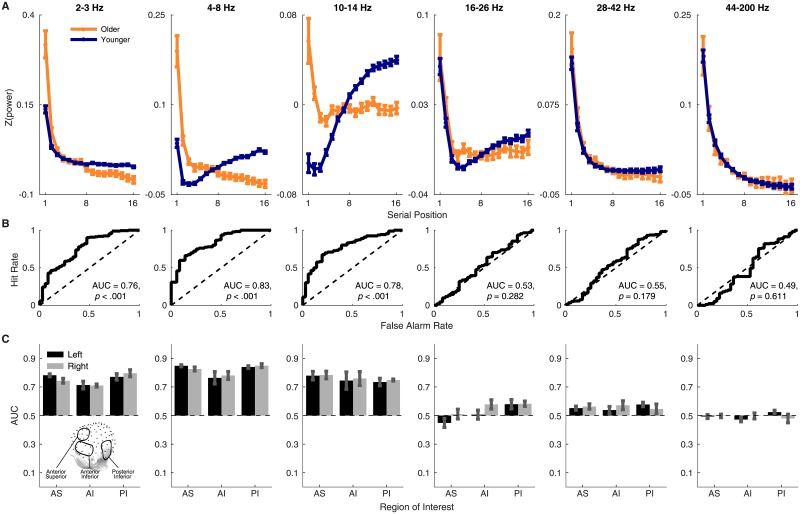
Age differences in spectral power gradients. A: Spectral power in six frequency bands across serial positions for younger adults versus older adults. Error bars are one standard error of the mean. B: ROC curves created by varying the threshold value of Δ_*EEG*_ (the change from the power level at the first serial position to the average power of the last 5 items) used to classify a subject as a younger or older adult. Significance was assessed by comparing the observed AUC value with a null distribution created by permuting Δ_*EEG*_ values across subjects 50000 times and running the analysis on each permuted dataset, with a strict Bonferoni correction to control *α* across the six comparisons, any AUC with *p* < (0.05/6) = 0.008 is significant. Note that the y-axis scale differs across panels (see the supplemental material for a version of the figure that uses a common scale for each band). C: AUC values from electrodes within six regions of interest (see insert of head map for locations). Here, error bars are 95% confidence intervals.

Fig 1A shows the gradient of spectral power across serial positions in six frequency bands. For younger adults, these gradients are in close agreement with those found in previous work [21]. In the 16–26 Hz, 28–42 Hz, and 44–200 Hz bands, both younger and older adults show high initial power followed by a rapid decline across serial positions, with little age difference. By contrast, the 2–3 Hz, 4–8 Hz, and 10–14 Hz bands all show clear age differences. Just as at higher frequencies, older adults exhibit a steep decline in power across serial positions at lower frequencies, but younger adults exhibit a shallower decline (in the 2–3 Hz band) or a net increase across serial positions (in the 4–8 Hz and 10–14 Hz bands). That is, older adults show higher power than younger adults early in a study list, but the age difference reverses for late-list items.

To determine if these neural gradients reliably predict age, we began by condensing the gradients into a single number for each subject by computing the change from the power level at the first serial position to the average power of the last 5 items:

$$\begin{array}{c} \Delta_{EEG}=\frac{\sum_{i=k}^{LL}SP_{i}}{LL-k+1}-SP_{1}, \end{array}$$ (1)

where *SP*_*i*_ is power during the *i*^*th*^ list item, *LL* is the total number of items in a list (here *LL* = 16), and *k* is the first item included in the late-item average (*k* = 5 for the analyses reported here). We then tested whether Δ_*EEG*_ distinguishes older from younger adults by examining receiver operating characteristic (ROC) curves created by varying the criterion value of Δ_*EEG*_ used to classify a subject as older if they are above the criterion and younger if they are below. To create the ROC for a given band, we started with a very high criterion value of Δ_*EEG*_ such that a younger adult is never misidentified as an older adult (i.e., zero false alarm rate) but older adults are also never correctly classified as older adults (i.e., zero hit rate) and then gradually decrease the criterion, tracing out a curve that shows how hit and false alarm rates change until the criterion is so low that all subjects are classified as older adults (i.e., perfect hit rate but also a 100% false alarm rate). Area under the curve (AUC) can be computed as a measure of sensitivity, with higher values indicating more sensitivity to age group and values near 0.5 indicating the measure is uninformative as to age group. The ROCs and AUCs (Fig 1B) show that the 2–3 Hz, 4–8 Hz, and 10–14 Hz gradients were all highly reliable biomarkers of age group. Significance was assessed by finding where the AUC for the actual ROC curves lay in a null AUC distribution formed by permuting Δ_*EEG*_ across subjects 50000 times and computing a ROC for each permuted dataset. The bottom row of Fig 1 shows the results of the ROC analysis conducted separately for six regions of interest (three areas each on the left and right sides: an anterior superior area, an anterior inferior area, and a posterior inferior area) commonly used in scalp EEG studies [19, 22, 23]. The results revealed that for the frequency bands that showed a whole-head effect, the effect was also present across all regions of interest.

**Fig 2:**
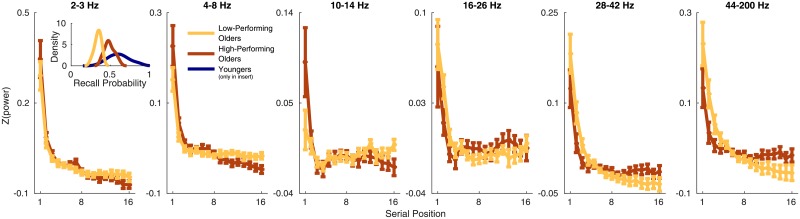
Spectral power in six frequency bands across serial positions for older adults with recall probabilities above (high-performing) versus below (low-performing) the older adult median. Error bars are one standard error of the mean. The insert in the first panel shows kernel density estimates of the distributions of overall probability of recall values for each group. Note that the y-axis scale differs across panels.

How do these age differences in neural dynamics relate to age age differences in memory ability? To explore this question, we conducted a median split analysis comparing the older adults with the highest memory scores to the older adults with the lowest memory scores (see the insert in the first panel of Fig 2). Previously analyzed free recall data, including studies that have far less data/subjects than our data set, have been shown to be highly reliable measures of individual differences that predict a variety of factors including age, IQ, memory ability, and clinical variables [24–32] suggesting that free recall is good measure of differences in memory ability between sub-groups of older adults. As shown in Fig 2, these subgroups showed distinct neural gradients.

In the 2–3 Hz, 4–8 Hz, and 10–14 Hz bands, the older adults with the largest memory impairments showed neural gradients that were more similar to the younger adult pattern of shallowly decreasing (2–3 Hz) or gradual increasing (4–8 Hz and 10–14 Hz) power across serial positions. That is, the best performing older adults looked *least* like younger adults at the neural level. A similar situation is observed at higher frequencies. Young adults show a steep decrease in power in the 28–42 Hz and 46–200 Hz bands, as do the low-performing older adults. But the high-performing older adults show a shallower decrease. Again, the high-performing older adults depart most strikingly from the younger adult pattern of neural dynamics.

ROC analyses on Δ_*EEG*_ values, analogous to those reported in Fig 1, revealed that no individual frequency band reliably discriminated low-performing from high-performing older adults (.06 < *p* < .20). However, the younger adult pattern is not fully described by any individual frequency band, instead it is characterized by gradual increases across serial positions at 10–14 Hz and sharp decreases for higher frequencies. To capture this pattern, we computed the difference between Δ_*EEG*_ in each lower frequency band, *F*_*i*_, and the 46–100 Hz band:

$$\begin{array}{c} \Delta_{EEG_{F_{i}}}-\Delta_{EEG_{44-200Hz}}. \end{array}$$ (2)

**Fig 3:**
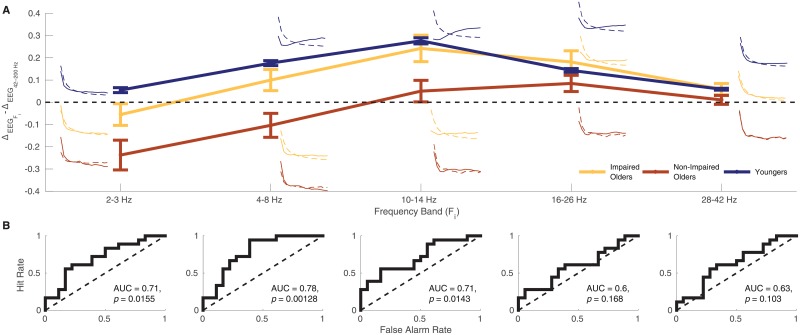
Spectral power distinguishes between low-performing and high-performing older adults. A: Mean values of $\Delta_{EEG_{F_{i}}}-\Delta_{EEG_{44-200Hz}}$ for the 2–3 Hz, 4–8 Hz, 10–14 Hz, 16–26 Hz, and 28–42 Hz bands for the younger adults and older adults with recall probabilities above (high-performing) the older adult median, and older adults below (low-performing) the older adult median. Error bars are one standard error of the mean. To ease interpretation of the $\Delta_{EEG_{F_{i}}}-\Delta_{EEG_{44-200Hz}}$ values, the small curves next to each data point show the full gradients across serial positions for the current frequency (*F*_*i*_, solid lines) and 44–200 Hz (dotted lines). $\Delta_{EEG_{F_{i}}}-\Delta_{EEG_{44-200Hz}}$ represents the difference in the rate of change of these two gradients. B: ROC curves created by varying the threshold value of $\Delta_{EEG_{F_{i}}}-\Delta_{EEG_{44-200Hz}}$ used to classify a subject as an low-performing versus a high-performing older adult. Significance was assessed by comparing the observed AUC value with a null distribution created by permuting $\Delta_{EEG_{F_{i}}}-\Delta_{EEG_{44-200Hz}}$ values across subjects 50000 times and running the analysis on each permuted dataset, with a strict Bonferoni correction to control *α* across the five comparisons, any AUC with *p* < (0.05/5) = 0.01.

Fig 3A compares this measure among younger adults, low-performing older adults, and high-performing older adults for each of the frequency bands. To ease interpretation the $\Delta_{EEG_{F_{i}}}-\Delta_{EEG_{44-200Hz}}$ values, the small curves next to each data point show the full gradients across serial positions for the current frequency (*F*_*i*_, solid lines) and 44–200 Hz (dotted lines). $\Delta_{EEG_{F_{i}}}-\Delta_{EEG_{44-200Hz}}$ represents the difference in the rate of change of these two gradients. At all frequencies, the low-performing older adults are numerically closer to the younger adult pattern than are the high-performing older adults. We conducted an ROC analysis on the ability of this measure to distinguish the two older adult subgroups. The measure for the 2–3 Hz, 4–8 Hz, and 10–14 Hz bands reliably discriminated low-performing from high-performing older adults (Fig 3B). It is critical to note that because this measure incorporates information about the 44–200Hz band into the lower frequency bands, it is impossible to attribute these effects to a single frequency band. They must be interpreted as the difference in rate of change across-serial positions of a given band versus the 44–200 Hz band. With this caveat in mind, we can see that larger deviation from the younger adult pattern of neural dynamics across an encoding episode is a biomarker of relatively preserved memory performance.

**Fig 4:**
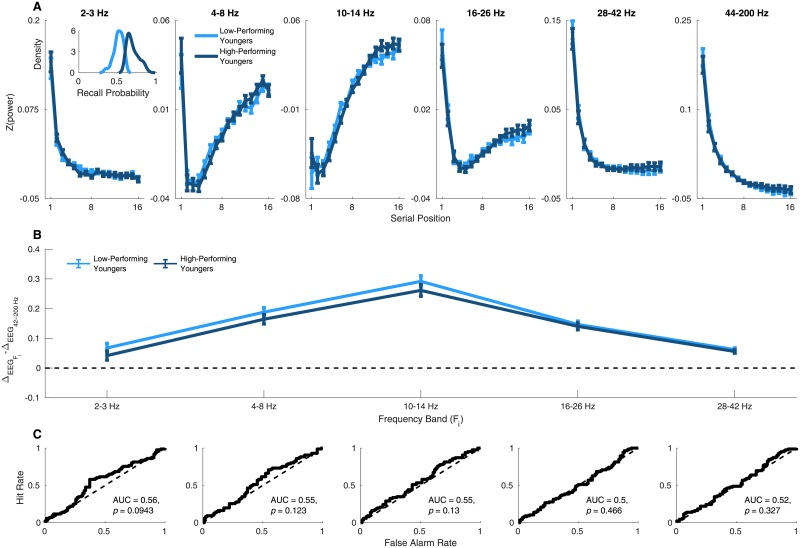
Spectral power *does not* distinguish between high- and low-performing younger adults. A: Mean values of $\Delta_{EEG_{F_{i}}}-\Delta_{EEG_{44-200Hz}}$ for the 2–3 Hz, 4–8 Hz, 10–14 Hz, 16–26 Hz, and 28–42 Hz bands for the younger adults with recall probabilities above the younger adult median, and younger adults below the median. Error bars are one standard error of the mean. $\Delta_{EEG_{F_{i}}}-\Delta_{EEG_{44-200Hz}}$ represents the difference in the rate of change of these two gradients. B: ROC curves created by varying the threshold value of $\Delta_{EEG_{F_{i}}}-\Delta_{EEG_{44-200Hz}}$ used to classify a subject as a high- versus a low-performing younger adult. Significance was assessed by comparing the observed AUC value with a null distribution created by permuting $\Delta_{EEG_{F_{i}}}-\Delta_{EEG_{44-200Hz}}$ values across subjects 50000 times and running the analysis on each permuted dataset, with a strict Bonferoni correction to control *α* across the five comparisons, any AUC with *p* < (0.05/5) = 0.01.

The reason the two sub-subgroups of older adults show different neural patterns may be that the high-performing older adults are compensating for age-related decline. Alternatively, it may be that the pattern exhibited by the low-performing older adults is simply a general feature of low-performing individuals that is not unique to age-related decline. We can test these possibilities by conducting the same median split analysis on the younger adult group. If the differences between the older adult sub-groups are due to age-related decline and compensation, then the younger adults, who of course have no age-related decline to compensate for, should *not* show these differences. Thus, we conducted the same median split analysis on on the younger adult group. The results of this analysis are presented in Fig 4 and confirm that whereas the neural patterns of low-performing versus high-performing older adults were quite distinct, the patterns of high-performing versus low-performing younger adults were quite similar. This suggests that the effects in the older adult group are specifically related to aging.

## Discussion

We found evidence of age differences in how neural activity changes while encoding a series of events. For both older and younger adults, high frequency oscillatory power (16–200 Hz) declined rapidly across events [21]. By contrast, power at lower frequencies showed marked age differences. Whereas older adults exhibited rapid power declines at both high and low frequencies, younger adults exhibited shallower decreases (2–3 Hz) and even rapid increases (10–14 Hz) at low frequencies. The rate and direction of change of the gradient at these low frequencies was a highly reliable biomarker of age, as revealed by ROC analyses. These results add neural dynamics across encoding periods to the growing list of age differences in electrophysiology [2, 33–37]. Intriguingly, older adults who performed best on the memory task showed the largest deviation from the younger adult pattern, particularly in the 4–14 Hz range. This finding complements previous work that has suggested that some aspects of age-related differences in processing compensates for, rather than contributes to, behavioral impairments [38–42].

Here, we provide evidence for the general hypothesis that there are age differences in the neural dynamics of encoding. We hope these preliminary results will be useful both in guiding basic science and in designing assessments to detect signs of memory impairment. To conclude, we highlight two important questions for future work and provide some speculations on promising answers.

The first question is which cognitive processes are linked to the observed age difference in neural dynamics? Two general categories of processes strike us as likely candidates: processes that become less efficient as the list progresses with time due to fatigue [5] and processes that ramp up as the list goes on such as rehearsing early items in the list. Although we have emphasized cognitive processes, such as attention and rehearsal, it is important, of course, to consider other possibilities. One possibility is that age-related anatomical changes such as a change in the ratio of white to gray matter may change how EEG signals propagate and thereby produce age differences in the patterns observed at the scalp. Future work, perhaps combining imaging techniques, will be needed to pursue these possibilities.

The second question is why would age differences in such processes compensate for, rather than exacerbate, memory impairment? In the case of fading efficiency, if older adults are aware they will fatigue across a list, it might make sense for them to strongly engage encoding processes for early items to ensure that at least some items are well-encoded. In the case of rehearsal, it is known that older adults are less likely to rehearse items [10], perhaps because they are impaired on the retrieval processes [4, 43] needed to think back to early list items [44]. If rehearsal is likely to fail, older adults may be well-served by instead focusing on encoding the current item. Indeed, alpha power (corresponding to the 10–14 Hz band used here) has been linked to holding more items in mind [45] and increases in 10–14 Hz power younger adults show across a list may be an index of elaborative encoding or rehearsal [21]. Alpha (and beta) power have also been linked to age-related differences in memory [46]. Therefore, the smaller increase of 10–14 Hz power in high-performing older adult group relative to the low-performing group may indicate that they are not attempting to engage in elaborative encoding or rehearsal. Future research should focus on determining whether the effects we have reported here do indeed reflect compensation and, if so, identifying which specific memory processes are involved.

## Supporting information

S1 Fig Age differences in spectral power in six frequency bands across serial positions for younger adults versus older adults. This presents the same data as Fig 1, but with each panel using the same y-axis scale. Error bars are one standard error of the mean.
(PDF)

## References

[1] Newson RS, Kemps EB. The nature of subjective cognitive complaints of older adults. International Journal of Aging Human Development. 2006;63(2):139–151. 10.2190/1EAP-FE20-PDWY-M6P1 17137031

[2] Werkle-Bergner M, Freunberger R, Sander MC, Lindenberger U, Klimesch W. Inter-individual performance differences in younger and older adults differentially relate to amplitude modulations and phase stability of oscillations controlling working memory contents. NeuroImage. 2012;60(1):71–82. 10.1016/j.neuroimage.2011.11.071 22178810

[3] Stark SM, Yassa MA, Stark CEL. Individual differences in spatial pattern separation performance associated with healthy aging in humans. Learning & Memory. 2010;17(6):284–288. 10.1101/lm.1768110 20495062 PMC2884287

[4] Healey MK, Kahana MJ. A Four–Component Model of Age–Related Memory Change. Psychological Review. 2016;123(1):23–69. 10.1037/rev0000015 26501233 PMC5067022

[5] Tulving E, Rosenbaum RS. What do explanations of the distinctiveness effect need to explain? In: Hunt RR, Worthen JB, editors. Distinctiveness and Memory. New York, NY: Oxford University Press; 2006 p. 407–423.

[6] Naveh-Benjamin M. Adult-Age Differences in Memory Performance: Tests of an Associative Deficit Hypothesis. Journal of Experimental Psychology: Learning, Memory, and Cognition. 2000;26:1170–1187. 10.1037//0278-7393.26.5.1170 11009251

[7] Hasher L, Zacks RT. Working memory, comprehension, and aging: A review and a new view In: Bower GH, editor. The psychology of learning and motivation: Advances in research and theory. San Diego: Academic Press; 1988 p. 193–225.

[8] Benjamin AS. Representational Explanations of “Process” Dissociations in Recognition: The DRYAD Theory of Aging and Memory Judgments. Psychological Review. 2010;117(4):1055–1079. 10.1037/a0020810 20822289 PMC3045270

[9] Healey MK, Kahana MJ. Age-Related Changes in the Dynamics of Memory Encoding Processes Provide a Biomarker of Successful Aging. Submitted. Submitted;.

[10] Ward G, Maylor EA. Age-related deficits in free recall: The role of rehearsal. Quarterly Journal Of Experimental Psychology. 2005;58A(1):98–119. 10.1080/02724980443000223 15881293

[11] Pergola G, Danet L, Pitel AL, Carlesimo GA, Segobin S, Pariente J, et al The regulatory role of the human mediodorsal thalamus. Trends in cognitive sciences. 2018;22(11):1011–1025. 10.1016/j.tics.2018.08.006 30236489 PMC6198112

[12] Katzman R, Brown T, Fuld P, Peck A, Schechter R, Schimmel H. Validation of a short Orientation-Memory-Concentration Test of cognitive impairment. The American Journal of Psychiatry. 1983;140(6):734–739. 10.1176/ajp.140.6.734 6846631

[13] Steyvers M, Shiffrin RM, Nelson DL. Word association spaces for predicting semantic similarity effects in episodic memory In: Healy AF, editor. Cognitive Psychology and its Applications: Festschrift in Honor of Lyle Bourne, Walter Kintsch, and Thomas Landauer. Washington, DC: American Psychological Association; 2004.

[14] Healey MK, Long NM, Kahana MJ. Contiguity in Episodic Memory. Psychonomic Bulletin & Review. 2019;. 10.3758/s13423-018-1537-3 PMC6529295 30465268

[15] Bell AJ, Sejnowski TJ. An information-maximization approach to blind separation and blind deconvolution. Neural Computation. 1995;7(6):1129–1159. 10.1162/neco.1995.7.6.1129 7584893

[16] Nolan H, Whelan R, Reilly R. FASTER: fully automated statistical thresholding for EEG artifact rejection. Journal of neuroscience methods. 2010;192(1):152–162. 10.1016/j.jneumeth.2010.07.015 20654646

[17] Delorme A, Makeig S. EEGLAB: an open source toolbox for analysis of single-trial EEG dynamics. Journal of Neuroscience Methods. 2004;134:9–21. 10.1016/j.jneumeth.2003.10.009 15102499

[18] Onton J, Makeig S. Information-based modeling of event-related brain dynamics. Progress in brain research. 2006;159:99–120. 10.1016/S0079-6123(06)59007-7 17071226

[19] Long NM, Kahana MJ. Modulation of task demands suggests that semantic processing interferes with the formation of episodic associations. Journal of Experimental Psychology: Learning, Memory, and Cognition. 2017;43(2):167–176. 10.1037/xlm0000300 27617775 PMC5290071

[20] Nyhus E, Curran T. Functional role of gamma and theta oscillations in episodic memory. Neuroscience & Biobehavioral Reviews. 2010;34(7):1023–1035. 10.1016/j.neubiorev.2009.12.014 20060015 PMC2856712

[21] Sederberg PB, Gauthier LV, Terushkin V, Miller JF, Barnathan JA, Kahana MJ. Oscillatory correlates of the primacy effect in episodic memory. NeuroImage. 2006;32(3):1422–1431. 10.1016/j.neuroimage.2006.04.223 16814568

[22] Weidemann CT, Mollison MV, Kahana MJ. Electrophysiological correlates of high-level perception during spatial navigation. Psychonomic Bulletin & Review. 2009;16(2):313–319. 10.3758/PBR.16.2.313 19293100 PMC2704578

[23] Curran T, Friedman W. ERP old/new effects at different retention intervals in recency discrimination tasks. 2004;8:107–120. 10.1016/j.cogbrainres.2003.09.006 14736570

[24] Kahana MJ, Howard MW, Zaromb F, Wingfield A. Age dissociates recency and lag recency effects in free recall. Journal of Experimental Psychology: Learning, Memory, and Cognition. 2002;28:530–540. 10.1037//0278-7393.28.3.530 12018505

[25] Wahlheim CN, Huff MJ. Age differences in the focus of retrieval: Evidence from dual-list free recall. Psychology and Aging. 2015;30(4):768 10.1037/pag0000049 26322551 PMC4679636

[26] Polyn SM, McCluey JD, Morton NW, Woolard AA, Luksik AS, Heckers S. Temporal context and the organisational impairment of memory search in schizophrenia. Cognitive neuropsychiatry. 2015;20(4):296–310. 10.1080/13546805.2015.1031372 25861879

[27] Pajkossy P, Keresztes A, Racsmány M. The interplay of trait worry and trait anxiety in determining episodic retrieval: The role of cognitive control. The Quarterly Journal of Experimental Psychology. 2017;70(11):2234–2250. 10.1080/17470218.2016.1230142 27603582

[28] Gibson BS, Healey MK, Gondoli DM. Modeling the Effect of Attention-Deficit Hyperactivity Disorder on Episodic Memory. Journal of Abnormal Psychology. 2019;.

[29] Sahakyan L, Kwapil TR. Moving Beyond Summary Scores: Decomposing Free Recall Performance to Understand Episodic Memory Deficits in Schizotypy. Journal of Experimental Psychology: General. 2018;Advance online publication. 10.1037/xge0000401 29369644

[30] Healey MK, Crutchley P, Kahana MJ. Individual differences in memory search and their relation to intelligence. Journal of Experimental Psychology: General. 2014;143(4):1553–1569. 10.1037/a0036306 24730719 PMC4128018

[31] Sederberg PB, Miller JF, Howard WH, Kahana MJ. The temporal contiguity effect predicts episodic memory performance. Memory & Cognition. 2010;38(6):689–699. 10.3758/MC.38.6.689 20852233

[32] Spillers GJ, Unsworth N. Variation in working memory capacity and temporal–contextual retrieval from episodic memory. Journal Experimental Psychology: Learning, Memory and Cognition. 2011;37(6):1532–1539. 10.1037/a0024852 21823812

[33] Caplan JB, Bottomley M, Kang P, Dixon RA. Distinguishing rhythmic from non-rhythmic brain activity during rest in healthy neurocognitive aging. NeuroImage. 2015;112:341–352. 10.1016/j.neuroimage.2015.03.001 25769279 PMC4408255

[34] Sander MC, Werkle-Bergner M, Lindenberger U. Amplitude modulations and inter-trial phase stability of alpha-oscillations differentially reflect working memory constraints across the lifespan. NeuroImage. 2012;59(1):646–654. 10.1016/j.neuroimage.2011.06.092 21763439

[35] Roca-Stappung M, Fernández T, Becerra J, Mendoza-Montoya O, Espino M, Harmony T. Healthy aging: relationship between quantitative electroencephalogram and cognition. Neuroscience letters. 2012;510(2):115–120. 10.1016/j.neulet.2012.01.015 22266305

[36] Voytek B, Kramer MA, Case J, Lepage KQ, Tempesta ZR, Knight RT, et al Age-Related Changes in 1/f Neural Electrophysiological Noise. The Journal of Neuroscience. 2015;35(38):13257–13265. 10.1523/JNEUROSCI.2332-14.2015 26400953 PMC4579381

[37] Zanto TP, Toy B, Gazzaley A. Delays in neural processing during working memory encoding in normal aging. Neuropsychologia. 2010;48(1):13–25. 10.1016/j.neuropsychologia.2009.08.003 19666036 PMC2794969

[38] Buckner RL. Memory and executive function in aging and AD: multiple factors that cause decline and reserve factors that compensate. Neuron. 2004;44(1):195–208. 10.1016/j.neuron.2004.09.006 15450170

[39] Gutchess A, Welsh RC, Hedden T, Bangert A, Minear M, Liu L, et al Aging and the neural correlates of successful picture encoding: frontal activations compensate for decreased medial-temporal activity. Journal of Cognitive Neuroscience. 2005;17(1):84–96. 10.1162/0898929052880048 15701241

[40] Zimmerman S, Hasher L, Goldstein D. Cognitive Ageing: a Positive Perspective In: Kapur N, editor. The Paradoxical Brain. Cambridge, U. K.: Cambridge University Press; 2011 p. 130–150.

[41] Lighthall NR, Huettel SA, Cabeza R. Functional compensation in the ventromedial prefrontal cortex improves memory-dependent decisions in older adults. The Journal of Neuroscience. 2014;34(47):15648–15657. 10.1523/JNEUROSCI.2888-14.2014 25411493 PMC4236396

[42] Daselaar SM, Iyengar V, Davis SW, Eklund K, Hayes SM, Cabeza RE. Less wiring, more firing: low-performing older adults compensate for impaired white matter with greater neural activity. Cerebral Cortex. 2015;25(4):983–990. 10.1093/cercor/bht289 24152545 PMC4366614

[43] Wingfield A, Kahana MJ. The dynamics of memory retrieval in older adults. Canadian Journal of Experimental Psychology. 2002;56:187–199. 10.1037/h0087396 12271749

[44] Laming D. An improved algorithm for predicting free recalls. Cognitive Psychology. 2008;57:179–219. 10.1016/j.cogpsych.2008.01.001 18329010

[45] Jensen O, Gelfand J, Kounios J, Lisman JE. Oscillations in the alpha band (9-12 Hz) increase with memory load during retention in a short-term memory task. Cerebral Cortex. 2002;12:877–882. 10.1093/cercor/12.8.877 12122036

[46] Guran CNA, Herweg NA, Bunzeck N. Age-related decreases in the retrieval practice effect directly relate to changes in alpha-beta oscillations. Journal of Neuroscience. 2019;39(22):4344–4352. 10.1523/JNEUROSCI.2791-18.2019 30902871 PMC6538864

