# Supplementary material for: Age-related differences in the temporal dynamics of spectral power during memory encoding
Source: PLoS One. 2020 Jan 16;15(1):e0227274. doi: 10.1371/journal.pone.0227274 (PMC6964832; doi:10.1371/journal.pone.0227274)
Supplement: S1 Fig — This presents the same data as Fig 1, but with each panel using the same y-axis scale. Error bars are one standard error of the mean. (PDF) [file pone.0227274.s001.pdf]

# Supplemental Methods for: Age-related differences in the temporal dynamics of spectral power during memory encoding

M. Karl Healey
Michigan State University

Michael J. Kahana
University of Pennsylvania

# Supplemental Methods for: Age-related differences in the temporal dynamics of spectral power during memory encoding

Figure 1 shows spectral power across output positions for each sub-group using a common y-axis scale for all frequency bands. This figure is intended to complement Figure 1 in the paper which show the same data with with varying scales to best visualize the data within each band.

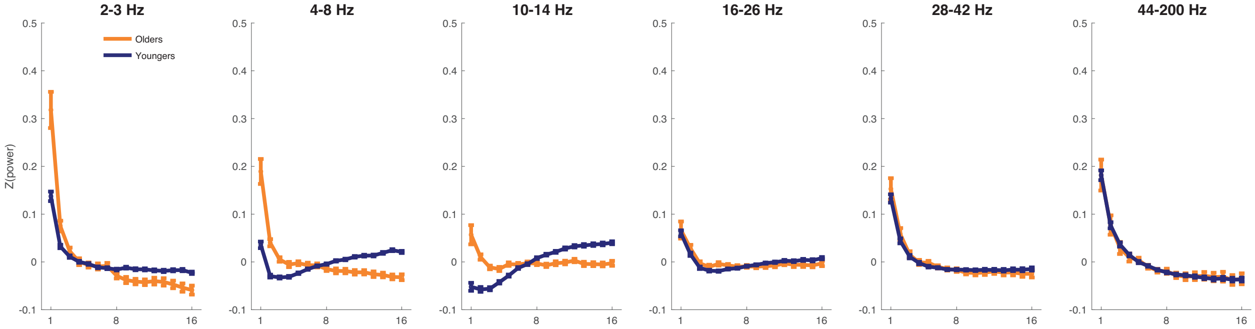


*Figure 1*. Age differences in spectral power in six frequency bands across serial positions for younger adults versus older adults. Error bars are one standard error of the mean.
